# Patient delay in cancer diagnosis: what do we really mean and can we be more specific?

**DOI:** 10.1186/1472-6963-14-387

**Published:** 2014-09-12

**Authors:** Christina Mary Dobson, Andrew James Russell, Greg Paul Rubin

**Affiliations:** School of Medicine, Pharmacy & Health, Durham University, Durham, UK; Department of Anthropology, Durham University, Durham, UK

**Keywords:** Delay, Cancer, Early diagnosis, Help-seeking, Symptom appraisal

## Abstract

**Background:**

Early diagnosis is a key focus of cancer control because of its association with survival. Delays in diagnosis can occur throughout the diagnostic pathway, within any one of its three component intervals: the patient interval, the primary care interval and the secondary care interval.

**Discussion:**

A key focus for help-seeking research in patients with symptoms of cancer has been the concept of ‘delay’. The literature is plagued by definitional and semantic problems, which serve to hinder comparison between studies. Use of the word ‘delay’ has been criticised as judgemental and potentially stigmatising, because of its implications of intent. However, the suggested alternatives (time to presentation, appraisal interval, help-seeking interval and postponement of help-seeking) still fail to accurately define the concept in hand, and often conflate three quite separate ideas; that of an interval, that of an unacceptably long interval, and that of a specific event which caused delay in the diagnostic process. We discuss the need to disentangle current terminology and suggest the term ‘prolonged interval’ as a more appropriate alternative. Most studies treat the patient interval as a dichotomous variable, with cases beyond a specified time point classified as ‘delay’. However, there are inconsistencies in both where this line is drawn, ranging from one week to three months, and how, with some studies imposing seemingly arbitrary time points, others utilising the median as a divisive tool or exploring quartiles within their data. This not only makes comparison problematic, but, as many studies do not differentiate between cancer site, also imposes boundaries which are not necessarily site-relevant. We argue that analysis of the patient interval should be based on presenting symptom, as opposed to pathology, to better reflect the context of the help-seeking interval, and suggest how new definitional boundaries could be developed.

**Summary:**

The word ‘delay’ is currently (conf)used to describe diverse conceptualisations of ‘delay’ and more mindful, and discerning language needs to be developed to enable a more sophisticated discussion. By stratifying help-seeking by presenting symptom(s), more accurate and informative analyses could be produced which, in turn, would result in more accurately targeted early diagnosis interventions.

## Background

Research into earlier diagnosis is a key focus for cancer control because of the growing evidence for an association between the time from symptom onset to diagnosis and both stage at diagnosis and subsequent survival [1–4]. There are several models of the diagnostic pathway that describe the stages from symptom onset to commencement of treatment, with these stages often being referred to as stages of delay [5–7]. The diagnostic pathway has been broken down into three component intervals: the period from symptom recognition to first consultation with a health care professional, generally a GP (termed by Olesen et al. [6] as patient delay), the period from first consultation with a health care professional to initiation of investigations for cancer related symptoms (doctor delay) and the period from initiation of investigations to commencement of treatment (system delay). Within the patient interval there are two component intervals; symptom appraisal (the period between detecting a bodily change and deciding that there is a need to discuss the symptoms with a health care practitioner) and help-seeking (the period from perceiving a need to discuss the symptoms with a health care practitioner to the first consultation) [7].

An understanding of the nature and duration of these intervals is crucial to research on cancer diagnosis, but also raises important questions about what constitutes ‘normality’ and where the temporal and behavioural boundaries of normality lie. We argue that the term ‘delay’ as currently used is both semantically and definitionally problematic and propose an alternative way of conceptualising variation in the patient interval based on symptom rather than eventual diagnosis.

A considerable body of research has sought to understand if, and how, a range of factors modify the patient interval, examining how the frequency, impact, and causes of such factors result in variation in its duration. Studies of the relationships between particular demographic characteristics and the duration of the patient interval have produced inconsistent findings [8–14], possibly because of the influence and diversity of barriers to presentation which have been shown to exist across demographic groupings [15]. Symptom misinterpretation is frequently reported, with patients believing their symptoms are the result of minor ailments [16–19], physical exertion [20, 21], stress [22], connected to pre-existing conditions [23], ageing [19, 24] or expected bodily changes [25]. Fear plays an important yet ambiguous role in help-seeking, acting as a prompt for some people and deterring presentation for others [26–29]. Fear can manifest itself as a fear of cancer or of the investigations and treatments associated with it [30, 31]. Fear of embarrassment and shame can also act as a barrier to presentation, particularly when symptoms are located in ‘private’ areas of the body [12, 20, 31]. Concern about wasting the doctor’s time, and appearing to be neurotic or hypochondriac, has been cited as a barrier to presentation [31, 32]. Some patients only report their symptoms during consultations for other conditions, or monitor their symptoms in order to accumulate ‘evidence’ to justify presentation [24, 31–34]. Social context has been shown to influence the timing of help-seeking, particularly the prioritisation of other life events [22, 26, 33–35]. Social networks are also thought to be influential, through the sanctioning of help-seeking [9, 36–38], and/or identification of symptoms [21, 39, 40].

Much of the public, and research, discourse around cancer diagnosis has been centred on the concept of ‘delay’. This body of research highlights the complexity of symptom appraisal and help-seeking processes, an issue which, it has been argued, has not been acknowledged in many previous studies [41].

Comparisons between studies of ‘delay’ within the patient interval are plagued with definitional and semantic inconsistencies [42]. We review these problems below. Our intention is not to jettison the term ‘delay’. We consider there to be great value in retaining the concept of delayed presentation as a function, or a tool, to guide future research, while recognising that help-seeking in particular occurs within the context of contending considerations, priorities and contexts. However, our conclusion is that ‘delay’ is better conceptualised based on symptomatology rather than diagnosis or eventual outcome.

## Discussion

### Specifying delay: semantic issues

There are common approaches within early diagnosis research for classifying the periods which constitute the diagnostic pathway, but there is less consistency in the language used to talk about it. Some authors refer to the periods within the diagnostic pathway as ‘phases of delay’ whereas others refer to them as ‘intervals’ [7, 43]. We will use the term patient interval, instead of phase, to refer to the period from symptom recognition to first consultation, and the terms appraisal interval and help-seeking interval to refer to its constituent parts. The word interval is also felt to be more precise in its scope than the much vaguer concept of a phase.

Use of the terms ‘delay’ and ‘patient delay’ is common but has faced criticism, as such terms are felt to be value laden, pejorative and judgemental [40, 44]. By labelling patients as ‘delayers’, there is felt to be an attribution of blame to the individual, which is potentially stigmatising. Critics of the use of the term ‘delay’ have suggested that other phrases, such as ‘appraisal interval’, ‘help-seeking interval’ or ‘time to presentation’ [40, 42] are preferable alternatives.

Although we agree that the language currently used is fundamentally flawed these proposed alternatives are also inaccurate, as they describe something which is conceptually different: that of a discrete interval within the diagnostic pathway. Most symptomatic patients will have an appraisal interval (the exceptions being those who have not identified a change in bodily sensations as abnormal), and all patients who consult a health care practitioner will also have a help-seeking interval, regardless of how long it takes them to consult. ‘Time to presentation’ is not clearly defined by those who have proposed the term. However, if we assume this phrase refers to the period from symptom onset to first presentation to a health care practitioner, which we have referred to as ‘the patient interval’, we are faced with the same issues inherent in the previous two suggestions. These three phrases all effectively describe intervals in the diagnostic pathway but tell us nothing about their nature, whether their length is necessary or undesirable or, if the latter, how their duration could have been reduced.

The term ‘postponement of help-seeking’ [29] has recently been used, which fits this purpose more precisely, as it clearly distinguishes a group who have taken longer to present. However, the use of the word ‘postpone’ still implies intention on the part of the patient (the Oxford English Dictionary entry for postpone states: *‘cause or arrange for (something) to take place at a time later than that first scheduled’*), which we know is often not the experience for patients in reality.

Current suggestions for alternative ways of referring to ‘delay’ appear to conflate three different concepts: that of an interval; that of an interval which is judged to have been unacceptably long; and that of an event which has caused a delay in a patient’s diagnosis.

We already have language which enables us to refer to discrete time periods, in the form of ‘intervals’, which are clearly defined. If we wish to treat the patient interval as a categorical variable in our analyses, we need to impose a boundary after which point symptom appraisal, help-seeking, or the patient interval in its entirety, are judged to be unacceptably long. This approach creates two groups within the dataset which have previously been conceived of as ‘delayers’ and ‘non-delayers’. These terms infer intent and we cannot suppose that these patients made a conscious decision to ‘delay’. We propose that more accurate and less value-laden terms to use when referring to this group are patients with an ‘acceptable’ or ‘prolonged’ interval (be it an appraisal, help-seeking or patient interval).

Dividing datasets into acceptable and prolonged intervals would enable us to examine the experiences of patients with prolonged intervals in greater detail. The purpose of such examinations would be to ascertain events which caused a delay in consulting a health care practitioner. Delays, in this context, are events within the help-seeking interval which interrupt the patient’s intended course of action, i.e. consultation. We refer exclusively to the help-seeking interval as, it has been argued that framing non-recognition of symptoms as an example of delay is merely an analytical construct based on biomedical understandings of symptomatology that bears little relation to individual experience and belief [45].

When identifying causes of delay, as well as being clear about our use of the word itself, we must be mindful as to how statements about causes are phrased. For instance, to say that *a patient delayed* because they were not able to get an appointment for four days after requesting one would be inaccurate, as it implies that an objective decision was made by the patient, and that they are at fault, when in reality the delay was beyond their control. However, if we said that *a lack of available appointments caused delay* within this patient’s help-seeking interval, we are shifting culpability from patient to context. This is particularly important because not all delays are avoidable. It is the avoidable delays which are of most interest, as these are the factors which have the greatest potential for modification.

To summarise, we may wish to describe data in its entirety, as the patient interval, or break it down further, into the appraisal interval and help-seeking interval. We may wish to understand how many patients present in a timely manner, and how many take an undesirably long time to present, requiring us to impose boundaries within our data. The cases which fall beyond the agreed cut off point would be best referred to as having prolonged intervals. To understand why these patients took longer to present we could explore individual cases, most appropriately using a qualitative approach, to ascertain the causes of delay in presentation whereby the focus is on the event, or context, as opposed to the individual.

### Specifying delay: methodological issues

There has been criticism that the approach commonly used to ascertain the duration of the patient interval (i.e. subtracting date of first symptom from date of first presentation) is too empirically grounded, as it assumes that there are objective, definitive dates when events occurred, which are readily collectable [41]. In reality, there is ambiguity in the individual, embodied experience of symptom development (i.e. from sensation to symptom), because of its grounding in social context [22], meaning that dates reported are more akin to interpreted estimates. Despite the subjectivity of the dates we collect in such studies, we believe that there is still value in using such data. However, we must be mindful that the dates provided are often ‘best estimates’ and will be influenced by the point that the patient has reached on the diagnostic pathway, as well as by recall bias. It is imperative that a theoretically and methodologically robust approach is adopted and best practice guidance should be followed. A good example is the Aarhus Statement [42], which states that the date of first symptom and the date of the first presentation should be consistently measured and described in order to facilitate comparison between studies.

Examinations of the patient interval often impose judgements as to the acceptability of its length. The duration of the patient interval is largely treated as a dichotomous variable, with a defined time point beyond which the interval has previously been classified as ‘delayed’. Many studies, following Pack and Gallo’s seminal work [46], adopt three months, or twelve weeks, as their definition of ‘delay’ [27, 30, 43, 47]. However, others have used time points of one month [48], thirty days [49], eight weeks [34], or patient intervals greater than the median [35, 50]. Not only does this make comparison between studies problematic [42], it also imposes an arbitrary judgement on timeliness of help-seeking across cancer sites that will have very different patterns of symptom development. It has been suggested that a preferable alternative would be to treat the patient interval as a continuous variable, with the median, as opposed to the mean, being presented because these data are usually positively skewed [15, 51]. However, using medians of study-specific datasets is also problematic, as it produces a relative, as opposed to absolute, judgement on the point signalling ‘delay’, making comparison between studies and generalisation from findings difficult.

Pedersen et al’s (2011) [37] approach was to generate quartiles from their patient interval data, and use the 25th and 75th quartiles to represent short and long ‘delay’ respectively. Although this approach is less indiscriminate than the selection of time points discussed above, it remains problematic as the quartiles were computed using a dataset which contained information for patients with a range of cancers. Producing categorisations of short and long ‘delay’ based upon data for multiple cancer sites can be misleading, since individual cancer sites have different biological and symptomatic progression, and also have a different likelihood of ‘delay’ [10, 52]. To assess ‘delay’ in patients with cancers which are known to be rapidly developing or more symptomatically troublesome using the same categories as for patients with cancers whose pathological development is more insidious, does not provide any greater insight into the appropriateness or otherwise of the length of the patient interval.

Another approach has been to attribute a label of ‘delay’ based upon the presenting symptom. Patients presenting with ‘red flag symptoms’ (i.e. change in a mole, a lump, or unusual bleeding) have been categorised as having ‘delayed’ if they did not present within one week of symptom onset, whilst patients reporting any other symptom have been categorised as having ‘delayed’ if it took them longer than four weeks to present [38, 53]. This approach is preferable, as it considers the nuances of symptom severity and development. However, the grouping of symptoms has been constructed within a biomedical framework; it is likely that an individual may not perceive all ‘red flag symptoms’ to be immediately threatening, or all ‘non-red flag symptoms’ to be of no immediate threat. The time points selected for these ‘alarm’ and ‘non-alarm’ symptoms are also quite arbitrary and, in fact, are not always clinically relevant, at least within the UK context. For instance, presenting with rectal bleeding of one week's duration would be unlikely to result in a referral to secondary care, based upon the NICE referral guidelines for suspected cancer [54].

Low et al. [50] investigated the patient interval by symptom, in relation to ovarian cancer, and found that anticipated length of help-seeking did vary by symptom, with women reporting the longest anticipated help-seeking for non-specific symptoms, such as fatigue and bloating, and shorter time to help-seeking for persistent abdominal pain. Although this study considers help-seeking by symptom, the analysis is based upon the responses of asymptomatic women to hypothetical situations. These responses are unlikely to mirror actual behaviour since such a methodology does not account for the numerous potential barriers to presentation discussed previously.

### A symptom-based approach to defining delay

Symptom appraisal and help seeking take place in specific social and temporal contexts and in response to the symptoms experienced as opposed to the condition ultimately diagnosed. Identification and analysis of prolonged patient intervals based upon presenting symptom, as opposed to pathology, would reflect more accurately the patient’s rationalisation and behaviour, which is ultimately based on their embodied experience of that symptom and perceptions of symptom severity.

Taking a generalised approach to their description, across cancer sites, or in relation to a particular cancer site, is problematic. Some patients would be characterised as ‘delayers’ when a period of watchful waiting may have been appropriate for the symptom they were experiencing. For instance, an acceptable period of watchful waiting for hoarseness would be much longer than an acceptable period of watchful waiting for haemoptysis (coughing up blood), yet if we examine the length of the patient interval by cancer site (i.e. lung), as opposed to presenting symptom these two examples are not currently differentiated.

Symptoms, even those termed alarm symptoms, have different predictive risks for cancer [55]. Campaigns to raise public awareness of cancer symptoms have been predicated largely on a clinical view about the importance of responding promptly to alarm symptoms, rather than on insights into which alarm symptoms are associated with less prompt action on the part of the patient [56]. Examination of the patient interval by symptom could produce a more useful basis upon which to consider areas for this type of intervention.

If we wish to analyse data by acceptable and prolonged intervals, definition of such boundaries is more easily achieved when focusing on individual symptoms. However, there is a lack of agreement among clinicians as to what constitutes an appropriate patient interval for particular symptoms and the clinical perspective often fails to take into account the patient’s understandings of symptoms and their implications. Defining new boundaries of prolonged intervals, based upon symptomatology, could be achieved through the analysis of secondary data sets, deriving quartiles from datasets of individual symptoms [37]. Such quartiles would act as a starting point from which to develop consensus around acceptable interval durations, seeking clinician and patient input to incorporate both biomedical and lay understandings in the definition of new boundaries.

Symptoms may develop concurrently and therefore the processes used to analyse multiple presenting symptoms need to be transparent [42]. Patients may identify two or more symptoms as arising simultaneously, or within a short period of time, reflecting the non-linear nature of symptom development and interpretation. Analytical approaches need to be mindful of this and should analyse the length of patient intervals both in relation to each symptom and combinations of symptoms. This would enable identification of individual, or combinations of, symptoms which are prone to prolonged help-seeking intervals.

Analysis of the patient interval by symptom, and identification of individual symptoms, or symptom clusters, which are associated with prolonged intervals, creates a foundation from which further research can seek to understand why such associations exist and to explore causes of delay more rigorously, with a view to reducing its effects in future.

## Summary

Current approaches to ‘delay’ within the patient interval, both in terms of linguistic definition and categorisation, are inconsistent and often atheoretical. Researchers in the field of early cancer diagnosis need to be more mindful of the terms they use, in particular ‘delay’, and consider their nuances and implications. Stratifying categorisation of prolonged intervals by symptom would result in more accurate and informative analyses of timely and prolonged symptom appraisal and, or, help-seeking. Results of such analyses can then function as starting points, from which we can attempt to understand barriers to presentation from a perspective more akin to that of an individual’s experience, i.e. one that is symptom-based as opposed to disease-based. Such an approach would not only be of relevance within the field of cancer, but could also be extrapolated to other conditions as well. In this way, we may be able to more accurately target interventions to address the obstacles faced by individuals most in need of support to facilitate their earlier presentation.

### Ethics statement

No ethical approval was sought, or obtained, as this discussion piece is conceptual, and not based on any specific study.
